# Amlodipine and the Successful Management of Post-Electroconvulsive Therapy Agitation

**DOI:** 10.1155/2016/3962491

**Published:** 2016-02-03

**Authors:** Ali Shahriari, Maryam Khooshideh, Mahdi Sheikh

**Affiliations:** ^1^Department of Anesthesiology, Roozbeh Hospital, Tehran University of Medical Sciences, Tehran 1333715914, Iran; ^2^Department of Obstetrics and Gynecology, Arash Hospital, Tehran University of Medical Sciences, Tehran 1653915981, Iran; ^3^Maternal, Fetal and Neonatal Research Center, Tehran University of Medical Sciences, Tehran 1419733141, Iran

## Abstract

Electroconvulsive therapy (ECT) is a highly effective nonpharmacologic treatment for the management of depression and some other psychiatric disorders. Post-ECT agitation occurs in up to 12% of ECT treatments and is characterized by motor restlessness, irritability, disorientation, and panic-like behaviors. The severity of post-ECT agitation ranges from mild and self-limited to serious and severe forms requiring prompt medical intervention to protect the patient and the medical staff. In severe agitation medical management may be necessary which consists of using sedative agents, either benzodiazepines or propofol. The side-effects of these sedative agents, especially in the elderly population, necessitate finding ways that could help the prevention of the occurrence of agitation after ECT treatments. We report a 68-year-old female with major depression who was referred for ECT. She experienced severe post-ECT agitation requiring medical intervention after all ECT treatments. Administering of oral amlodipine (5 mg) one hour before ECT treatment successfully prevented the occurrence of post-ECT agitation in this patient. We briefly discuss the possible underlying mechanisms and pathophysiology of amlodipine in the prevention of post-ECT agitation.

## 1. Introduction

Electroconvulsive therapy (ECT) is a nonpharmacologic treatment through inducing generalized seizures for therapeutic purposes, which is documented to be highly effective for the treatment of depression and some other psychiatric disorders [1]. By the use and progression of modern anesthesia and modified stimulation techniques the safety and tolerability of ECT have been dramatically increased [1, 2]. However ECT treatment is still associated with some complications ranging from mild headaches to memory problems and arrhythmia [2, 3]. Post-ECT agitation is another complication ranging from mild and self-limited to serious and severe forms requiring prompt medical intervention to protect the patient and the medical staff [2–5]. Post-ECT agitation occurs in 5%–12% ECT treatments and is characterized by motor restlessness, irritability, disorientation, and panic-like behaviors [2, 5]. When agitation is severe or prolonged medical management may be necessary which consists of using sedative agents, either benzodiazepines (midazolam or lorazepam) or propofol [5]. The side-effects of these sedative agents, especially in the elderly population [6], attracted the researchers attentions towards finding drugs that could help in the prevention of the occurrence of agitation after ECT treatments [3, 4].

We report a 68-year-old female with major depression and severe post-ECT agitation requiring medical intervention after all ECT treatments, which was successfully prevented by administering amlodipine one hour before ECT treatment.

## 2. Case Presentation

A 68-year-old female (65 kg) with treatment resistant major depression and unremarkable medical history was referred for ECT for the first time. Her medications before ECT course were citalopram, quetiapine, and carbamazepine. The patient underwent six sessions of ECT treatment with bilateral electrode placement and a stimulus dose of 40%–50% (approximately 216–270 mC) with the Thymatron DGx device (Somatics, LLC, Lake Bluff, IL), three times weekly. At each ECT session after physical examinations and performing electrocardiography (ECG) and monitoring the blood pressure (BP) and heart rate (HR) and pulse oximetry (SpO_2_) and preoxygenation, anesthesia was induced using propofol (80 mg), atropine (0.5 mg), and succinylcholine (35 mg) as paralytic agents. Ventilation was carried out by 100% oxygen using Mapleson breathing systems. Seizure duration was adequate for all treatments. Upon awakening after the first ECT session the patient became very agitated with a Richmond Agitation Sedation Scale (RASS) score of +2 to +3. Several people were required to restrain the patient, putting the staff and herself at risk of injury. Patient's agitation was treated with intravenous midazolam (5 mg). Postictal agitation was repeated after the second and third ECT sessions which responded well to midazolam. Before starting the fourth and fifth ECT sessions, the patient had an elevated BP compared to previous sessions; therefore the patient received oral amlodipine (5 mg) one hour before ECT treatment. Her BP before administering amlodipine was 140/90 mmHg, which fell to 120/80 mmHg before ECT treatment and remained the same after ECT. Interestingly after the fourth and fifth ECT treatment sessions the patient was calm upon awakening with no agitation. Before the sixth ECT treatment the patient had a BP of 120/80 mmHg; therefore amlodipine was not administered. As expected after the ECT treatment the patient became agitated upon awakening that lasted up to 60 minutes. The patient's depression significantly improved following six ECT sessions.

## 3. Discussion

The etiology and mechanism of post-ECT agitation are still not known. However the following controversial risk factors have been reported [2]: electrodes placement (unilateral versus bifrontal), lithium comedication, choice and dosing of anesthetic agents and muscle relaxants, and patients anxiety prior to ECT. Based on the available literature we postulate that the sudden and transient increase in cerebral blood flow might be one of the underlying mechanisms for the postictal agitation and headache associated with ECT treatments [7–9]; ECT treatment can cause cerebral blood flow to transiently increase to 300% of baseline measurements [7]; transient cerebral hyperperfusion has been shown to cause temporary agitation and delirium in patients undergoing surgical decompression of chronic subdural hematoma [8], as well as patients undergoing endarterectomy for carotid artery stenosis [9]. Additionally it is suggested that systemic hypertension (both preexisting and ECT induced) can increase the risk of agitation through promoting cerebral hyperperfusion [7, 8].

In our study we showed for the first time that administration of oral amlodipine one hour before ECT treatment might have preventive effects on the occurrence of post-ECT agitation. Although the exact mechanism is unknown, based on the available literature several mechanisms including the effects of calcium channel blockers (CCBs) on cerebral perfusion pressure (CPP) and central nervous system excitability might underlie the observed effect of amlodipine on prevention of post-ECT agitation. CPP is defined as the difference between mean arterial pressure (MAP) and intracranial pressure (ICP) (CCP = MAP − ICP) [10]. CCBs lower systemic hypertension, thus decreasing the MAP [11], and, by their effect on decreasing cerebral vascular resistance [12], they have been shown to slightly increase the ICP [11]. The net effect will be the decrease in CPP [11]; these effects might attenuate the sudden increase in cerebral blood flow [7], thus decreasing the risk of post-ECT agitation [7, 10–12].

Another underlying mechanism might be through the effect of amlodipine on calcium channels; inhibition of calcium entry into the calcium channels in the nerve terminals hyperpolarizes the presynaptic neuron resulting in a decrease in presynaptic norepinephrine release and inhibition of postsynaptic activity, thus the attenuation of central nervous system excitation [4].

In conclusion oral administration of 5 mg amlodipine one hour before ECT treatment might have preventive effects on the occurrence of post-ECT agitation especially in the elderly and hypertensive patients. However more reports are required to approve this finding and also to reveal the underlying mechanism before recommending the use of amlodipine before ECT treatments.
